# Upcycling Fruit and Vegetable Pomace Powders for Technological, Nutritional and Sensory Enhancement of Cookies

**DOI:** 10.17113/ftb.64.03.26.9485

**Published:** 2026-09-15

**Authors:** Selene Kozyra Bilicich, César Bruguezi, Nadia Lingiardi, Daniela Matjazic, Regina Formigli, Gisela Piccirilli, Roxana Verdini, Emilce Llopart, Marina Soazo

**Affiliations:** 1 Institute of Chemistry of Rosario (IQUIR), Suipacha 531, 2000 Rosario, Santa Fe, Argentina; 2 Department of Food and Environmental Sciences, Bromatology and Nutrition Area, Faculty of Biochemical and Pharmaceutical Sciences, National University of Rosario, Suipacha 531, 2000 Rosario, Santa Fe, Argentina; 3 National Scientific and Technical Research Council (CONICET), 2000 Rosario, Santa Fe, Argentina

**Keywords:** pomace, dietary fiber, bioaccessibility, polyphenols, antioxidant activity, sustainability

## Abstract

**Research background:**

Upcycling fruit and vegetable pomace into value-added bioproducts is an effective strategy to reduce food waste while enhancing the nutritional quality of processed foods. Cookies, typically high in sugar and fat, are good candidates for reformulation.

**Experimental approach:**

This study aims to evaluate an integrated reformulation strategy combining the incorporation of whole fruit and vegetable pomace with simultaneous sugar and fat reduction in cookies. Three whole pomaces from different fruit and vegetable blends were individually used as partial replacers for oil (25–50 %) and sugar (30–60 %). The chemical composition, technological attributes and sensory acceptance of reformulated cookies were determined. Total phenolic content and antioxidant capacity were quantified in digested samples to assess the influence of pomace incorporation on the bioaccessibility of bioactive compounds.

**Results and conclusions:**

Pomace incorporation markedly increased total, insoluble, and soluble fiber contents, with higher replacement yielding greater nutritional enhancement. Reformulated cookies also showed increased total phenolic content and antioxidant capacity in digested samples, indicating improved bioaccessibility of phenolic compounds. Technological parameters were moderately affected: hardness increased and spread ratio decreased with increasing pomace amount. Color changes occurred but remained within acceptable limits. Sensory evaluation by a trained panel revealed only slight reductions in crispness and overall liking at the highest incorporation of pomace, indicating that the sensory attributes of pomace-containing cookies were largely maintained. Therefore, formulations with intermediate pomace incorporation and moderate sugar and oil reductions (samples 25/30 and 50/30, where the first number denotes *w*(oil)_reduction_ and the second number *w*(sugar)_reduction_ in %) provided the best balance between technological quality, sensory attributes, and phenolic bioaccessibility, while higher replacement (sample 50/60) did not confer additional overall benefits.

**Novelty and scientific contribution:**

This study demonstrates that integrating fruit and vegetable pomace into cookie formulation with simultaneous sugar and fat reduction enables the development of a nutritionally enhanced product with moderate technological changes within acceptable quality ranges. The approach not only improves fiber content and phenolic bioaccessibility, but also converts processing residues into value-added bioproducts with functional significance. These findings provide a feasible pathway for designing healthier baked goods while promoting circularity and sustainability in food systems.

## INTRODUCTION

Cookies and other sweet baked goods are an integral part of dietary patterns worldwide, consumed across different age groups and socio-cultural contexts. Their popularity is largely driven by their appealing sensory attributes, relative affordability and extended shelf life, which have facilitated their incorporation into daily diets as common snack items in both household and commercial settings (*1*). However, conventional formulations are typically rich in sugar and fat, which contribute to high energy density and are associated with increased risks of overweight, obesity, type 2 diabetes, and cardiovascular diseases. In response to growing public health concerns, global authorities and the food industry have intensified efforts to reformulate processed foods to reduce the content of critical nutrients such as free sugars and saturated fats, while maintaining adequate technological quality. These strategies align with current consumer trends that increasingly demand products that are both health-promoting and sustainably produced (*2*).

Reformulating cookies poses technological and sensory challenges, since sugar and fat play multiple roles beyond sweetness and energy content, including structuring, texture development, spread during baking, and flavor release. Identifying alternative ingredients that can partially replace these components while maintaining acceptable product quality has therefore become a priority in food innovation (*3*).

Fruit and vegetable pomaces, by-products obtained after juice extraction, have recently gained attention as promising functional ingredients in bakery products. They are rich sources of dietary fiber, phenolic compounds, minerals and antioxidants, and their incorporation into baked goods can improve nutritional value while contributing to the circular economy and waste reduction (*4*). Pomaces from apple, pear, grapefruit and other fruits, have been used to enhance the fiber content and bioactive profiles of cookies, often without major negative impacts on sensory acceptance when used in moderate amounts (*5*–*7*). Moreover, valorizing whole pomace, without prior extraction of bioactive compounds, represents a sustainable and cost-effective approach to developing functional foods (*8*).

Despite growing interest in using fruit and vegetable pomaces as functional ingredients, most previous research has primarily focused on enriching baked products to increase dietary fiber and antioxidant contents, while keeping sugar and fat contents unchanged (*8*, *9*). These studies consistently report nutritional improvements, but also highlight the technological and sensory challenges associated with pomace incorporation (*10*, *11*).

However, the simultaneous incorporation of whole pomace with sugar and fat reduction has been scarcely addressed as a combined strategy. Many approaches rely on the isolation of specific fiber fractions to substitute sugar or fat, which entails additional processing steps and costs (*12*).

Furthermore, the impact of such reformulations on the gastrointestinal release of phenolics and antioxidant capacity has received little attention, despite its relevance for anticipating potential health benefits. To address these gaps, this study proposes an integrated cookie reformulation strategy that simultaneously reduces sugar and oil amounts relative to a recipe while incorporating whole fruit and vegetable pomaces as functional ingredients. The chemical composition, technological parameters, sensory properties and gastrointestinal bioaccessibility of antioxidant compounds in the reformulated cookies were thoroughly characterized. By combining multiple reformulation strategies and valorizing pomace without prior extraction of bioactive compounds, this work offers a holistic approach to enhancing the nutritional and sensory quality of cookies, while promoting the sustainable use of food processing by-products.

## MATERIALS AND METHODS

### Materials

Wheat flour type 000 (Molino Juan Semino S.A., Santa Fe, Argentina), refined sugar (David Rosental e Hijos S.A.I.C., Rosario, Argentina), high-oleic sunflower oil (Cañuelas, Buenos Aires, Argentina), instant oats (Morixe, Buenos Aires, Argentina), baking powder (Kraft Foods, Buenos Aires, Argentina), vanilla essence (Alicante, Rosario, Argentina), potable water, and fresh mature orange, carrot, pineapple, lemon, ginger, beetroot, apple, pear and radish (a cultivar purchased from a local market) were used. Folin-Ciocalteu reagent, 6-hydroxy-2,5,7,8-tetramethylchroman-2-carboxylic acid (Trolox), 2,2′-azino-bis(3-ethylbenzthiazoline)-6-sulphonic (ABTS) acid, α-amylase (from porcine pancreas), pepsin (from porcine gastric mucosa), pancreatin (from porcine pancreas) and bile (porcine extract) were purchased from Sigma-Aldrich®, Merck (Buenos Aires, Argentina). All other reagents were of analytical grade.

### Juice formulation and pomace collection

Three pressed juices were prepared based on commercially available recipes. The fruit and vegetable combinations were as follows (in %): juice 1 contained orange 56, carrot 18, pineapple 18, lemon 7 and ginger 1; juice 2 consisted of carrot 33, beetroot 28, apple 26 and lemon 13; and juice 3 of apple 43, pear 40, orange 16 and radish 1. The raw materials were washed with potable water, cut and/or peeled when necessary, and then alternately processed in a juicer (Max Juicer PE-SJ15; Peabody, Guangzhou, PR China). The solid residues retained in the filter were collected and hereafter referred to as pomaces P1, P2 and P3, corresponding to juices 1, 2 and 3, respectively. Each process was repeated until approx. 5 kg of wet pomace was obtained. The pomaces were dried in an electric convection oven (Deli-4; Moretti, Buenos Aires, Argentina) at 100 °C until the moisture content was below 15 %, then cooled to room temperature, ground using a grinder (TDMC; Tecnodalvo, Santa Fe, Argentina) and vacuum-packed with a packaging machine (VAC 510-E; Neovac, Santa Fe, Argentina) until further analyses.

### Pomace characterization

Moisture content was determined by oven drying to constant mass at 105 °C according to AOAC method 925.10 (*13*), using a forced-air drying oven (CHRI; Tecnodalvo, Santa Fe, Argentina). Ash content was measured by incineration at 550 °C following AOAC method 923.03 (*14*), using a muffle furnace (HM5; Tecnodalvo). Protein content was determined by the Kjeldahl method (AOAC 920.87) (*15*), based on total nitrogen quantification. Samples were digested using a digestion unit (DK6; VELP Scientifica, Usmate, Italy) and subsequently distilled using a distillation unit (UDK129; VELP Scientifica). A nitrogen-to-protein conversion factor of 6.25 was applied. Dietary fiber fractions, including total dietary fiber (TDF), insoluble dietary fiber (IDF), and soluble dietary fiber (SDF), were determined according to the enzymatic–gravimetric method (AOAC 991.43) (*16*), using a commercial assay kit (K-TDFR; Megazyme, Wicklow, Ireland), based on enzymatic digestion followed by gravimetric quantification. Reducing, non-reducing and total sugars were determined by the Fehling–Causse–Bonnans titrimetric method, based on the reduction of copper(II) ions under alkaline conditions (AOAC 974.06) (*17*). All analyses were performed in triplicate.

### Cookie formulation

Control cookies, previously optimized by our research group (data not shown), were formulated with wheat flour, sugar, water, high-oleic sunflower oil, oats, baking powder and vanilla essence. Based on the control formulation, the oil and sugar contents were reduced at two mass fractions: 25 and 50 % for oil, and 30 and 60 % for sugar. The cookies were designated as 25/30, 25/60, 50/30 and 50/60 corresponding to each reduction mass fraction, respectively (Table 1). These reductions were compensated by replacing the corresponding ingredients with each pomace (P1, P2 or P3). The remaining ingredients were kept constant, as in the control cookies. This resulted in four different cookie formulations for each pomace (CP1, CP2 and CP3).

**Table 1:** Formulation of the different cookie samples according to oil and sugar mass fraction reduction

	Cookie sample				
Ingredient	Control	25/30	25/60	50/30	50/60
	*w*(ingredient)/%				
Wheat flour	52	52	52	52	52
Pomace	0	12	19	16	23
Oil	19	14	14	10	10
Sugar	24	17	10	17	10
Oat	4	4	4	4	4
Baking powder	0.5	0.5	0.5	0.5	0.5
Vanilla essence	0.5	0.5	0.5	0.5	0.5

25/30, 25/60, 50/30 and 50/60=cookies with reduced mass fractions of oil by 25 and 50 % and sugar by 30 and 60 %, respectively

For dough development, sugar, prehydrated oats, vanilla essence and sunflower oil were mixed with an electric mixer (HR7922/90; Philips, Buenos Aires, Argentina) for 2 min, until a creamy consistency was achieved. The previously sifted dry ingredients were then gradually incorporated, adding water as needed to obtain a homogeneous dough. The dough was rested for 15 min, then rolled out and cut into circular shapes with a cookie cutter. The cookies were baked in a preheated electric convection oven (Zonda 5-tray; Met Zonda S.A., Buenos Aires, Argentina) at 180 °C and then cooled to room temperature before analysis.

### Cookie characterization

#### Chemical composition

The proximate composition was determined according to AOAC methods: moisture (925.10) (*13*), ash (923.03) (*14*), protein (920.87, CF=6.25) (*15*), total fat determined by solvent extraction (Soxhlet method) (920.85) (*18*), TDF, IDF and SDF (991.43) (*16*). Carbohydrates were calculated by difference, according to the following equation:


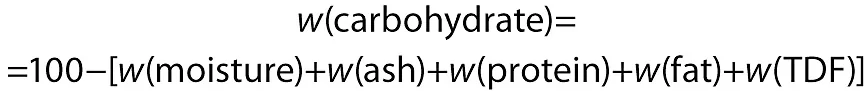
 /1/

Each analysis was conducted in triplicate. Energy density (kcal/g) was estimated from proximate composition using the Atwater factors (4 kcal/g for proteins and carbohydrates, and 9 kcal/g for lipids).

#### Physical and technological parameters

The physical and technological properties of the cookies (diameter, thickness, spread ratio, texture and color) were evaluated as described by Santuccione *et al*. (*19*). Cookie dimensions were measured with a caliper (Ruhlmann 200 mm, Buenos Aires, Argentina), and the spread ratio was calculated as the diameter-to-thickness ratio. Texture was determined by a three-point bending test using a motorized testing machine (Multitest 2.5-d; Mecmesin Ltd., Slinfold, UK) at a crosshead speed of 180 mm/min, with hardness defined as the maximum force at fracture. Color parameters (L*, a* and b*) were obtained by digital image analysis under standardized illumination, and the total color difference (ΔE*) was calculated to assess perceptible differences between samples:


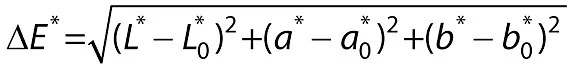
 /2/

where *L*_0_*, *a*_0_* and *b*_0_* are the values for the 25/30 cookie formulation, which had the lowest oil and sugar reductions, while L*, a* and b* are the values for the formulations with higher reductions (25/60, 50/30 and 50/60, respectively).

All measurements were performed in quintuplicate (*N*=5).

#### *In vitro* gastrointestinal digestion of cookies

The *in vitro* gastrointestinal digestion of cookie samples (CP1, CP2 and CP3 and their reduced formulations) was performed according to the standardized INFOGEST 2.0 protocol (*20*), simulating the oral, gastric and intestinal phases. Simulated salivary, gastric and intestinal fluids (1.25×) were prepared from stock saline solutions and adjusted to pH=7.0, 3.0 and 7.0, respectively. Fresh enzyme solutions (amylase, lipase, pepsin and pancreatin) and bile salts were prepared immediately before digestion. Aliquots of 5 g of cookies were subjected to sequential digestion at 37 °C under constant agitation (2 min oral phase; 120 min gastric and intestinal phases), with pH monitored and adjusted as required. After digestion, samples were centrifuged (VT3216; Cavour, Córdoba, Argentina) at 1500×*g*, and the soluble fractions were collected and stored at −20 °C until analysis. Enzyme blanks were included, and all experiments were performed in duplicate.

#### Antioxidant activity of digested samples

Antioxidant capacity was determined using the ABTṠ^+^ decolorization assay according to Llopart *et al*. (*21*), with minor modifications. The ABTṠ^+^ radical was generated by reacting 7 mM ABTS with 2.45 mM dipotassium peroxydisulfate in the dark at room temperature for 16 h and then diluted with a *φ*(ethanol,water)=50 % to an absorbance of 0.70±0.02 at 730 nm. For the assay, 60 µL of the soluble fraction obtained after *in vitro* digestion were mixed with 840 µL of the ABTṠ^+^ working solution and incubated in the dark for 25 min. Absorbance was measured at 730 nm using a UV/Vis spectrophotometer (model 330; Metrolab S.A., Buenos Aires, Argentina). Antioxidant capacity was quantified using a Trolox calibration curve (0.01–0.1 mg/mL) and expressed as mg of Trolox equivalents (TE) per 100 g of sample. All measurements were performed in triplicate.

#### Total phenolic content of digested samples

Total phenolic content (TPC) was determined using the Folin-Ciocalteu method according to Llopart *et al.* (*21*), with slight modifications. An aliquot of 40 µL of the soluble fraction obtained after *in vitro* digestion was mixed with 2 mL of Folin-Ciocalteu reagent (1:10) and 1 mL of 7.5 % Na_2_CO_3_, and incubated at 40 °C for 10 min. Absorbance was measured at 760 nm using a UV/Vis spectrophotometer (model 330; Metrolab S.A.). TPC was quantified using a gallic acid calibration curve (0.025–0.700 mg/mL) and expressed as mg gallic acid equivalents (GAE) per 100 g of sample. All determinations were performed in triplicate.

#### Sensory analysis

A quantitative descriptive analysis (QDA) was conducted with eight panelists (3 male and 5 female), aged between 30 and 50, trained in tasting baked goods, recruited from the Faculty of Biochemical and Pharmaceutical Sciences at the National University of Rosario (Rosario, Argentina). All participants were informed about the study procedures and signed a written informed consent. Training included aroma, texture and taste recognition and discrimination, as well as descriptive assessments of baked products and pomace obtained from fruit and vegetable blends. A consensus list of attributes was subsequently established (Table S1). The attributes assessed were color, hardness, sweet aroma, oil aroma, foreign aroma, sweet taste, oil taste, foreign taste, chewiness, graininess, and overall acceptability, with “foreign aroma” and “foreign taste” referring to the pomace used in the formulations. A 10-cm unstructured line scale, anchored at 1 cm from each end, was used for each descriptor, with anchors indicating minimum and maximum intensities. Panelists marked the perceived intensity on the scale, and values were recorded as the distance (cm) from the left end (*22*). Evaluations were carried out in individual booths, in accordance with the International Organization for Standardization (ISO 11132:2021; ISO 8589:2007) (*23*, *24*).

### Statistical analysis

Data were analyzed by ANOVA using Minitab® 18.1 (*25*) and expressed as mean value±standard deviation. Significant differences between formulations were identified using Tukey’s HSD test (p<0.05).

## RESULTS AND DISCUSSION

### Pomace recovery and characterization

The wet pomaces obtained showed an initial moisture mass fraction of 80–85 %, in line with values previously reported for orange (*26*), apple (*27*) and pear pomaces (*28*), as well as for carrot (*29*) and beetroot (*30*). The high water content and the presence of fermentable sugars underline the need for drying or immediate processing to avoid microbial spoilage and reduce transport volume (*26*). After drying, the final moisture of the three pomaces fell below 7 % (Table 2). The ash mass fraction of the pomaces varied markedly among samples, which may be attributed to the predominant raw materials in each pomace. P1 presented intermediate values consistent with those reported for orange pomace, around 3-5 % (*12*, *31*). The high ash mass fraction in P2 may be related to its high amount of carrot, with reported values from 5.29 to 5.89 % depending on variety (*32*), and beetroot, which showed ash mass fraction around 4 % (*33*). Conversely, P3 exhibited the lowest ash mass fraction, in agreement with the generally lower mineral content reported for apple and pear pomaces (*31*). Regarding protein mass fraction, higher values were found for pomaces containing a greater proportion of carrot and beetroot, in line with previous reports indicating that vegetable pomaces tend to be richer in protein than fruit-derived ones (*32*, *34*, *35*). Previous studies have reported that carrot pomace powders can contain appreciable protein mass fractions (around 6–9 %, depending on source and drying conditions) (*32*), while beetroot pomace may reach up to 11 % (*33*). In contrast, fruit pomaces such as apple and orange are typically lower in protein but richer in pectin and soluble fiber (*34*, *35*).

**Table 2:** Pomace characterization

Component	P1	P2	P3
	*w*(component)/%		
Moisture before drying	(83.2±0.3)^a^	(80.1±0.4)^b^	(80.6±0.4)^b^
Moisture after drying	(6.86±0.02)^a^	(4.54±0.01)^c^	(5.4±0.1)^b^
Ash	(4.11±0.05)^b^	(6.00±0.03)^a^	(1.94±0.05)^c^
Protein	(6.2±0.2)^b^	(9.5±0.2)^a^	(2.9±0.3)^c^
TDF	(48±5)^a^	(42±1)^a^	(40±4)^a^
SDF	(16±3)^a^	(11±2)^a^	(10±3)^a^
IDF	(32±1)^a^	(31±1)^a^	(30±3)^a^
Reducing sugars	(20±1)^b^	(9±1)^c^	(28±1)^a^
Non-reducing sugars	(10±2)^a^	(8±1)^a^	(7±1)^a^
Total sugars	(30±1)^a^	(17±1)^b^	(34±3)^a^

Different letters in superscript in the same row indicate significant differences among pomaces (P1, P2 and P3). TDF, SDF and IDF=total, soluble and insoluble dietary fiber, respectively

Table 2 also summarizes the contents of total dietary fiber (TDF), insoluble dietary fiber (IDF), and soluble dietary fiber (SDF). As expected, TDF mass fractions reflect the similar fiber contribution of the source fruits and vegetables (*36*). These results are consistent with previous studies, which reported TDF mass fractions ranging from 20–30 % for carrot pomace (*32*), 50–70 % for other fruit pomaces (*31*), and up to 80 % for apple pomace (*37*). In addition, all pomaces provided both IDF and SDF fractions, reinforcing their nutritional relevance due to the diverse functional properties and health benefits associated with these fiber types (*38*).

Regarding sugar mass fractions, P1 and P3 showed similar, while P2 lower values (Table 2). These differences reflect the sugar composition of the raw materials used. P3, mainly derived from apple and pear, had higher sugar mass fractions (10.4 and 9.8 g, respectively) (*36*). In contrast, P2 contained apple, beetroot and carrot, whose sugar mass fractions are 10.4, 6.8 and 4.7 g, respectively (*36*). P1 was primarily composed of orange (9.35 g) (*36*), which explains why its total sugar mass fraction was similar to that of P3. In terms of sugar profile, significant differences were observed in reducing sugar content: P3 had the highest value, followed by P1, while P2 had the lowest, consistent with the composition of the corresponding raw materials.

### Chemical composition of the formulated cookies

The chemical composition of the cookie formulations varied with both the type of pomace (P1, P2 and P3) used and the mass fraction of fat and sugar reduction (Table 3). Moisture content increased in formulations with higher amounts of pomace and in those with greater sugar reduction. This behavior is consistent with the high water-holding capacity of pomace fiber: fruit and vegetable pomaces are rich in soluble and insoluble dietary fiber that absorb and retain water, increasing product moisture content. This effect is associated with hydrogen bonding between hydrophilic polysaccharides and water molecules, which limits water mobility and promotes greater water retention during baking (*39*). A similar pattern was reported in a study on wheat flour cookies enriched with 5–15 % apple pomace, which showed that higher amounts of this pomace significantly increased water absorption capacity, consequently raising the moisture content of the cookies (*40*). Additionally, slightly higher moisture values were observed in the formulations with the same mass fractions of oil and sugar reduction but prepared with P1 and P3; these results can be attributed to the higher moisture content determined in these components compared to that of P2. A trend towards higher ash mass fraction was observed in CP1 and CP2 formulations with increasing amounts of pomace, suggesting that higher amounts of pomace incorporation contribute additional minerals to the product. Differences in ash mass fraction between pomace types for the same formulation likely reflect the intrinsic mineral composition of each pomace, as described above, corresponding to minerals naturally present in the original fruits and vegetables and further concentrated during the pomace production process (*41*). Additionally, substituting oil with pomace reduces the added lipid fraction; therefore, the observed decreases in total fat in higher fat-reduction formulations are consistent with compositional expectations, and this trend was observed regardless of the type of pomace used. As for protein content, the data show minimal variation in protein mass fraction across the different formulations. When comparing pomace types, cookies containing P3 consistently exhibited the lowest protein mass fractions among analogous formulations. These differences reflect the inherent compositional variability of the pomaces, suggesting that the extent of protein variation in the final product depends on both the type of pomace used and its substitution amount.

**Table 3:** Chemical composition of the cookies formulated with different pomaces

Sample	Cookie formulation	*w*(moisture)/%	*w*(ash)/%	*w*(protein)/%	*w*(total fat)/%	*w*(TDF)/%	*w*(IDF)/%	*w*(SDF)/%	*w*(CH)/%	Energy density /(kcal/g)
CP1	25/30	(11.0±0.1)^dA^	(0.9±0.1)^dB^	(7.1±0.2)^aB^	(15.0±0.4)^aA^	(5.5±0.1)^cB^	(2.4±0.3)^cB^	(3.1±0.1)^abA^	(60.5±0.2)^aA^	(4.06±0.03)^aA^
	25/60	(13.9±0.3)^cA^	(1.20±0.01)^bB^	(7.4±0.1)^aB^	(14.1±0.4)^aA^	(8.4±0.1)^bA^	(4.5±0.1)^bAB^	(3.9±0.3)^abA^	(55.0±0.8)^bA^	(3.70±0.01)^bB^
	50/30	(15.4±0.8)^bA^	(1.10±0.01)^cB^	(7.3±0.1)^aB^	(9.6±0.1)^bA^	(8.5±0.4)^bA^	(4.1±0.2)^bAB^	(4.3±0.2)^aA^	(58.1±0.4)^abA^	(3.48±0.02)^cB^
	50/60	(18.2±0.1)^aA^	(1.33±0.03)^aB^	(7.0±0.3)^aB^	(7.0±0.9)^cA^	(9.8±0.3)^aA^	(6.0±0.4)^aA^	(3.9±0.1)^abB^	(56±2)^bA^	(3.17±0.03)^dB^
CP2	25/30	(8.4±0.1)^dB^	(1.2±0.1)^dA^	(7.9±0.1)^aA^	(14.6±0.1)^aA^	(6.8±0.0)^bcAB^	(4.1±0.3)^bcA^	(2.7±0.3)^bcB^	(61.2±0.1)^aA^	(4.07±0.01)^aA^
	25/60	(12.5±0.1)^bB^	(1.50±0.01)^bA^	(8.2±0.2)^aA^	(14.4±0.1)^aA^	(7.7±0.8)^bA^	(5.4±0.6)^abA^	(2.3±0.3)^cB^	(55.7±0.6)^bA^	(3.82±0.04)^bA^
	50/30	(11.5±0.1)^cB^	(1.30±0.01)^cA^	(8.1±0.1)^aA^	(10.1±0.1)^bA^	(7.9±0.1)^bA^	(3.7±0.2)^cB^	(4.1±0.1)^aA^	(61.1±0.3)^aA^	(3.68±0.01)^cA^
	50/60	(16.3±0.1)^aB^	(1.6±0.01)^aA^	(7.40±0.01)^bA^	(8.1±0.2)^cA^	(10.3±0.3)^aA^	(6.7±0.1)^aA^	(3.6±0.4)^abB^	(56.4±0.5)^bA^	(3.28±0.02)^dA^
CP3	25/30	(10.9±0.1)^dA^	(0.70±0.01)^cC^	(7.1±0.2)^aB^	(14.2±0.3)^aA^	(7.5±0.3)^bcA^	(4.6±0.2)^cA^	(2.8±0.1)^bB^	(59.7±0.7)^aA^	(3.95±0.03)^aB^
	25/60	(14.7±0.5)^bA^	(0.90±0.01)^aC^	(6.8±0.2)^abC^	(13.5±0.4)^aA^	(8.2±0.2)^bA^	(5.6±0.3)^bA^	(2.5±0.1)^bB^	(56.0±0.4)^bA^	(3.72±0.01)^bB^
	50/30	(13.8±0.2)^cA^	(0.80±0.01)^bC^	(6.4±0.4)^abC^	(9.4±0.2)^bA^	(8.3±0.1)^bA^	(5.8±0.2)^bA^	(2.4±0.3)^bB^	(61.4±0.7)^aA^	(3.55±0.01)^cB^
	50/60	(18.2±0.1)^aA^	(0.80±0.01)^bC^	(5.7±0.5)^bC^	(7.8±0.2)^cA^	(11±2)^aA^	(6.8±0.1)^aA^	(4.7±0.1)^aA^	(56±1)^bA^	(3.17±0.07)^dB^

CP1, CP2 and CP3=cookies formulated with pomace 1 (P1), 2 (P2) and 3 (P3), TDF, IDF and SDF=total, insoluble and soluble dietary fiber, respectively, CH=carbohydrates. 25/30, 25/60, 50/30 and 50/60=cookies with reduced mass fractions of oil by 25 and 50 % and sugar by 30 and 60 %, respectively. Different lowercase letters in superscript in the same column indicate significant differences among cookies formulated with the same pomace. Different uppercase letters in superscript in the same column indicate significant differences between samples with the same reduction level, but different pomace types

The analysis of carbohydrate content revealed that, as expected, it is strongly affected by the formulation. Cookies formulated with greater sugar reductions consistently showed lower total carbohydrate mass fractions than those with smaller sugar reductions. This finding is consistent with the role of sugar as an important contributor to the carbohydrate fraction in baked goods. No significant differences were observed among pomaces, indicating that the fiber-rich by-products themselves did not markedly alter the overall carbohydrate fraction in the final cookies. This suggests that, at the substitution mass fractions used, pomace incorporation mainly redistributed carbohydrates towards non-digestible fiber rather than increasing total carbohydrate content.

The dietary fiber content of the cookie formulations varied significantly with the amount of pomace incorporation, with greater reductions in added fat and sugar leading to higher mass fractions of total, insoluble and soluble fiber. This outcome is consistent with the previously described compositional profile of by-products, which are recognized sources of dietary fiber, with the greatest mass fraction of insoluble fiber (Table 2). Previous studies have reported similar outcomes, where the incorporation of 2.5–10 % berry or maqui pomaces markedly increased the fiber content of cookies, indicating a substantial enhancement of the nutritional quality of bakery products (*11*, *41*). From a nutritional perspective, the fiber enrichment achieved in the present formulations may help meet the recommended daily intake of 25 g per day (*42*). This is particularly relevant given that cookies are commonly consumed snack foods, offering a practical vehicle for increasing dietary fiber, which is widely recognized for its role in promoting gastrointestinal health and reducing the risk of chronic diseases (*42*).

The energy density of the cookie formulations decreased significantly as sugar and oil were progressively reduced and replaced with pomaces. The most pronounced reductions were observed at the greatest replacement mass fraction (50/60). This effect can be attributed to the lower caloric contribution of pomaces, which are rich in dietary fiber, and to the reduction in fat and sugar, the main energy sources in cookies. From a nutritional perspective, energy-dense foods are often linked to excessive caloric intake and an increased risk of overweight and obesity, so reformulating bakery products can contribute to healthier dietary patterns and support public health recommendations aimed at reducing energy intake from ultra-processed foods (*2*). Although such reformulation strategies align with public health recommendations, their actual impact on energy intake depends on consumption context and portion size.

### Physical and technological characterization of the formulated cookies

#### Color

The color parameters and visual appearance of the different formulations are shown in Fig. 1. The incorporation of fruit and vegetable pomaces significantly influenced the color attributes of the cookies, with the effect varying depending on the type of pomace used. For CP1 formulations, a progressive reduction in lightness was observed as pomace amount increased, with values ranging from the highest in the 25/30 sample to the lowest in the 50/60 sample, confirming a darkening effect. This trend is consistent with the findings of Andrejko *et al.* (*43*), who reported that the inclusion of carrot and apple pomace in oat cookies significantly decreased L* values while increasing a* and b* components due to the presence of carotenoids, which impart reddish and yellowish tones. In our case, the increases in both a* and b* with the addition of P1 may also be related to the influence of carotenoid-rich residues, in line with earlier reports showing that β-carotene from carrot remains sufficiently stable during baking to affect final product color (*44*). Moreover, color differences (ΔE*>5) in all CP1 formulations (Table 4) indicate perceptible variations to consumers, similar to the perceptibility thresholds reported by Andrejko *et al*. (*43*), where *ΔE* values above 5 were associated with visible differences when carrot pomace was added to cookies.

**Fig. 1:**
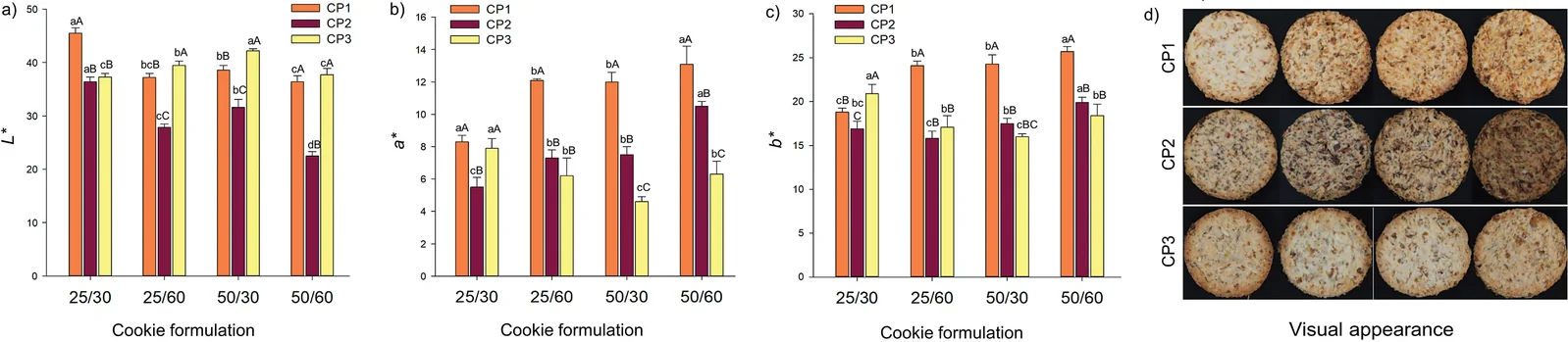
Color parameters: a) L*, b) a*, c) b* and d) visual appearance of cookies formulated with pomace 1 (CP1), pomace 2 (CP2) and pomace 3 (CP3). 25/30, 25/60, 50/30 and 50/60=cookies with reduced mass fractions of oil by 25 and 50 % and sugar by 30 and 60 %, respectively. Different lowercase letters indicate significant differences between formulations with the same pomace. Different uppercase letters indicate significant differences between samples with the same reduction level, but different pomace types

**Table 4:** Physical and technological parameters of the cookies formulated with different pomaces

						

Sample	Cookie formulation	ΔE*	Hardness/N	*d*/mm	*δ*/cm	Spread ratio d/*δ*
CP1	25/30	-	(28±2)^aA^	(4.9±0.1)^aA^	(1.00±0.01)^bAB^	(4.9±0.1)^aAB^
	25/60	(10.6±0.9)^ab^	(21±3)^bA^	(4.70±0.01)^bAB^	(1.10±0.03)^aA^	(4.40±0.03)^bAB^
	50/30	(9.7±1.6)^b^	(29 ±3)^aA^	(4.70±0.04)^bAB^	(1.10±0.03)^aAB^	(4.20±0.04)^bB^
	50/60	(12.5±1.4)^a^	(22±2)^bA^	(4.70±0.07)^bA^	(1.00±0.03)^bB^	(4.80±0.07)^aA^
CP2	25/30	-	(34±5)^aA^	(4.90±0.03)^aA^	(0.90±0.01)^bB^	(5.50±0.03)^aA^
	25/60	(9.0±1.3)^b^	(24 ±4)^bA^	(4.8±0.1)^bcA^	(1.10±0.02)^aA^	(4.50±0.04)^bA^
	50/30	(5.4±1.0)^c^	(36±5)^aA^	(4.8±0.2)^bA^	(1.00±0.02)^aB^	(4.8±0.1)^bA^
	50/60	(15.2±1.3)^a^	(20±3)^bA^	(4.7±0.1)^cA^	(1.00±0.03)^aB^	(4.7±0.1)^bA^
CP3	25/30	-	(33±7)^aA^	(4.7±0.2)^aB^	(1.10±0.01)^bA^	(4.4±0.2)^aB^
	25/60	(3.9±2.7)^b^	(23±3)^bA^	(4.7±0.2)^aAB^	(1.10±0.03)^bA^	(4.30±0.05)^aB^
	50/30	(7.3±1.1)^a^	(32±4)^aA^	(4.7±0.3)^aAB^	(1.20±0.02)^aA^	(4.10±0.06)^aB^
	50/60	(3.0±0.9)^b^	(23±3)^bA^	(4.6±0.1)^aB^	(1.10±0.01)^bA^	(4.40±0.01)^aB^

CP1, CP2 and CP3=cookies formulated with pomace 1 (P1),2 (P2), and 3 (P3). 25/30, 25/60, 50/30 and 50/60=cookies with reduced mass fractions of oil by 25 and 50 % and sugar by 30 and 60 %, respectively. Color variation (Δ*E*) relative to sample 25/30. Different letters in superscript in the same column indicate significant differences among cookies formulated with the same pomace. Different uppercase letters in superscript indicate significant differences between samples with the same reduction level, but different pomace types

In CP2, L* values decreased with higher pomace addition, while both a* and b* increased, resulting in an intense reddish-pink hue. The most pronounced differences were observed in the 50/60 formulation, which exhibited the highest contrast compared with 25/30. These results agree with Mitrevski *et al*. (*33*), who demonstrated that increasing beetroot powder (15–25 %) in biscuits significantly reduced L* and elevated a*, producing cookies with a darker and more reddish appearance due to the strong pigmentation effect of betalains. Similarly, Kruczek *et al.* (*9*) reported that betalains not only withstand thermal processing but also intensify red coloration in bakery matrices. Our results, showing ΔE* values above 5 for all CP2 formulations (Table 4), reinforce the strong visual impact of beet residues as dominant drivers of bakery product color.

In contrast, CP3 displayed a different pattern. L* values increased up to the 50/30 formulation, while a* and b* decreased, suggesting a dilution of the typical brownish shades generated during baking. However, the 50/60 formulation deviated from this trend, showing minimal color difference relative to 25/30. This subtle effect is likely due to the relatively neutral pigmentation of apple and pear residues. Similar observations were reported by Naseem *et al*. (*40*), who showed that substituting wheat flour with apple pomace powder (5–15 %) decreased L* only moderately and induced slight changes in a* and b*, depending on the substitution amount. Andrejko *et al.* (*43*) also highlighted that apple pomace has less effect on cookie coloration than carrot, corroborating our findings for CP3.

In addition to pigment composition, the sugar content of the pomaces likely contributed to the observed color changes. Although P2 contained less total and reducing sugars, its high betalain content may have dominated the color profile, leading to pronounced decreases in L* and increases in a* and b*. In contrast, P1 and P3 supplied not only pigments but also higher mass fractions of total and reducing sugars, which could promote Maillard reaction and caramelization during baking (*45*). In CP1, this combination intensified browning and color intensity, while in CP3, the relatively neutral pigments from apple and pear diluted the natural color development of the matrix, partially offsetting the darkening effect of Maillard reactions. This interplay may explain the non-linear trends observed for CP3, particularly in formulations with higher pomace amounts. Furthermore, the reduction of added sucrose (30 and 60 %) and its partial replacement by pomace-derived sugars may have further modulated Maillard reactions, highlighting the complex interaction between endogenous sugars and formulation adjustments in determining the final cookie color beyond the direct contribution of pigments.

#### Texture

Texture analysis revealed a clear trend among the different formulations, as shown in Table 4. Cookies with lower sugar reductions (25/30 and 50/30) showed significantly higher hardness values than formulations with greater sugar reductions (25/60 and 50/60), regardless of the type of pomace. These findings are consistent with previous studies reporting that sugar content contributes to cookie firmness and crispness by dissolving in the available water in the dough and, upon baking, recrystallizing and forming an amorphous glassy phase that strengthens the structure. This glassy sugar phase limits molecular mobility in the baked matrix, resulting in increased rigidity and hardness (*46*). However, no significant differences were observed between formulations with the same sugar content but different mass fractions of oil. Reducing oil mass fraction did not significantly affect cookie hardness in this study. This observation aligns with Sudha *et al.* (*47*), who found that fat reduction in soft dough cookies led to increased dough hardness but did not necessarily translate into harder baked products. The minimal impact of oil reduction on hardness in this study may be attributed to the compensatory effects of pomace addition, which could influence moisture content and overall texture more strongly than oil content alone. In particular, fiber–water interactions associated with pomace incorporation may have contributed, partially offsetting the expected effects of lipid reduction on texture.

#### Cookie diameter, thickness and spread ratio

Formulation and pomace type significantly influenced the technological parameters of the cookies (Table 4). Regarding diameter, the 25/30 formulation generally produced the largest cookies across pomace types. This behavior can be attributed to the higher sugar and oil contents, which may favor dough spreading during baking by enhancing system mobility and delaying structure setting (*46*). In CP1 and CP2, a consistent reduction in diameter was observed with greater oil and sugar reduction, with significantly lower values for 25/60, 50/30 and 50/60 formulations than for 25/30. In contrast, CP3 showed no significant differences among formulations, suggesting a compensatory effect of this pomace on dough spread, possibly linked to its fiber composition. Differences among pomace types were also evident: CP1 and CP2 yielded larger diameters than CP3, particularly in 25/30 and 50/60 formulations. Certain fibers can contribute to the liquid phase and soften the dough, thereby promoting expansion, while others can retain absorbed water and increase dough cohesiveness, limiting spreading (*48*).

Thickness was primarily influenced by formulation, tending towards slightly higher values at greater oil and sugar reduction, particularly in the 25/60 and 50/30 formulations of CP1 and CP2. However, these differences were not entirely consistent across pomace types. Thickness values of CP3 were generally higher than of CP2 in the 25/30 formulation, but the overall pattern remained variable. Spread ratio reflected the combined effects of diameter and thickness. The highest values were observed for CP2 with 25/30 formulation, consistent with its larger diameter and lower thickness. In most other cases, spread ratios decreased with greater reduction of oil and sugar, especially for CP1 and CP3, indicating reduced dough expansion during baking. CP3 consistently showed the lowest spread ratios, in agreement with its smaller diameters and relatively higher thickness.

Overall, the results confirm that cookie dimensions and spread behavior are strongly affected by the balance between fat, sugar and fiber in the formulation. However, the patterns observed were not always linear or consistent across pomace types, reflecting the complex interactions between ingredients during dough processing and baking. Similar variability has been reported in other studies on sugar and fat reduction, where differences in fiber source, water distribution and matrix composition can lead to divergent technological responses (*49*, *50*). These findings highlight that the technological behavior of reformulated cookies cannot be predicted solely from individual ingredient functions, but depends on the combined and sometimes compensatory effects arising from the specific formulation and fiber source used.

#### Antioxidant activity and total phenolic content in digested samples

Following *in vitro* gastrointestinal digestion, all formulations enriched with pomace (up to 19 % substitution) showed similarly high antioxidant activities, expressed as TE, ranging from approx. 3600 to 3900 mg/100 g of cookies (Fig. 2). Only the formulation with the greatest oil and sugar reduction (50/60 with 23 % pomace substitution) showed a slightly lower value, suggesting that excessive substitution may not further enhance and could even slightly limit antioxidant potential. This pattern suggests that, beyond an optimal substitution amount, excessive fiber addition can alter cookie microstructure, restricting antioxidant compound diffusion or extraction during digestion. Such non-linear behavior has been reported by Yagci *et al.* (*51*), who studied the effect of adding tomato pomace powder to extruded snacks. They found that increasing pomace from 0 to 20 % enhanced antioxidant capacity by up to 15 %, but no further improvement was observed at 20 %, indicating that higher pomace contents do not necessarily lead to proportional gains in antioxidant potential.

**Fig. 2:**
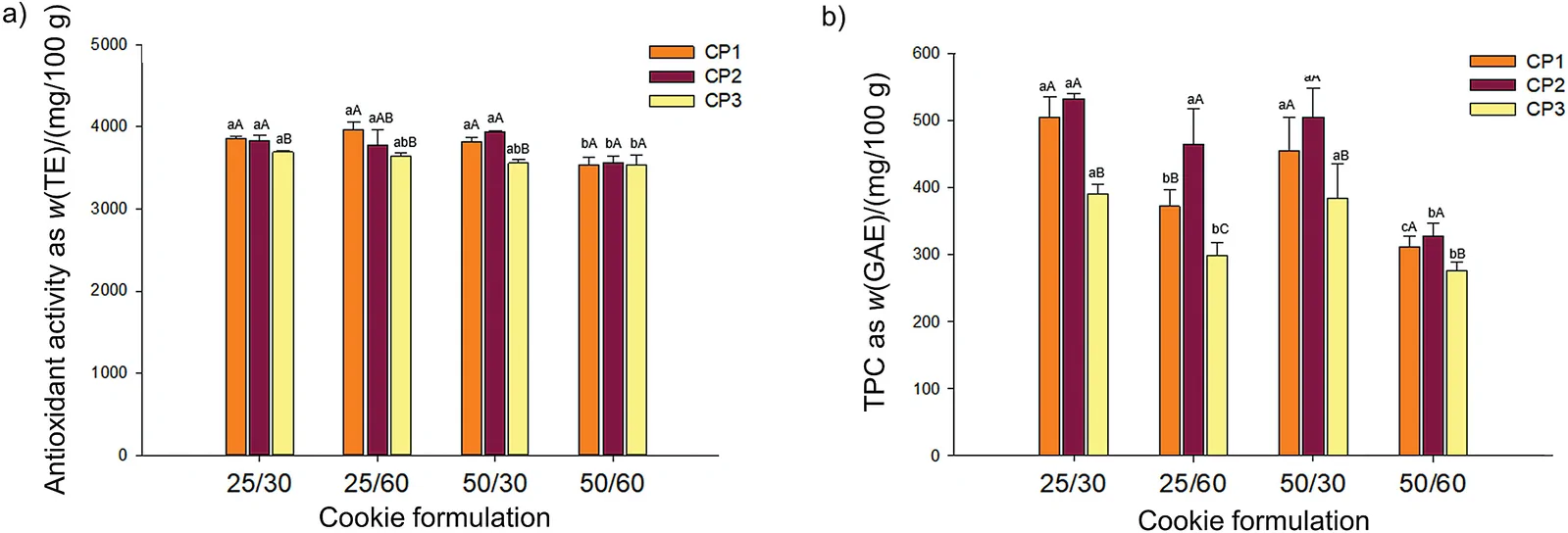
Results for: a) antioxidant activity and b) total phenolic content of cookies formulated with pomace 1 (CP1), pomace 2 (CP2), and pomace 3 (CP3) after *in vitro* digestion. 25/30, 25/60, 50/30 and 50/60=cookies with reduced mass fractions of oil by 25 and 50 % and sugar by 30 and 60 %, respectively. Different lowercase letters indicate significant differences between formulations with the same pomace. Different uppercase letters indicate significant differences between samples with the same reduction level, but different pomace types. TE=Trolox equivalents, GAE=gallic acid equivalents

When comparing pomace types, CP1 and CP2 consistently delivered the highest ABTS values, which likely reflect differences in pomace composition, including the nature and proportion of antioxidant compounds, as well as their interactions with the food matrix. Thus, pomaces containing more stable, readily releasable bioactives tended to show higher values, while those with phenolics more closely associated with fiber may exhibit slightly weaker post-digestive activity (*47*).

Consistent with these findings, total phenolic content (TPC) after digestion, expressed as GAE, ranged from approx. 200 to 500 mg/100 g (Fig. 2). The highest TPC values were found in formulations containing 12–17 % pomace (25/30 and 50/30), suggesting that moderate substitutions favor the retention or release of phenolic compounds during digestion. In contrast, higher pomace incorporation (19–23 % in 25/60 and 50/60) led to a decline in TPC, possibly because the higher fiber content limited phenolic accessibility. The observed differences among pomace types further emphasize the influence of their inherent compositional characteristics, including phenolic profile and compound stability, on their recovery and activity after digestion (*52*). Moreover, Rocchetti *et al*. (*53*) investigated the effect of the addition of *Moringa oleifera* L. leaf powder on the phenolic bioaccessibility of durum wheat fresh pasta. and found a non-linear trend between polyphenol bioaccessibility and increasing amounts of powder added to the formulation.

Taken together, the ABTS and TPC results could be related not only to the release of phenolic compounds bound to the fiber-rich matrix, as mentioned above, but also to their potential interactions with other matrix components, which may be influenced by the reductions in oil and sugar content. Lower lipid content may reduce the solubilization of lipophilic antioxidant compounds during digestion, while changes in sugar content can modify water availability, indirectly affecting phenolic release. Additionally, the formation of new antioxidant compounds during processing could further contribute to the observed activity. It should also be noted that the ABTS assay may detect other redox-active molecules beyond phenolics, which could partly explain the measured values. However, excessive fiber/pomace seems to limit extractability (*54*). Accordingly, these methods, widely accepted for estimating the antioxidant potential of digested food matrices, provide an overall measure and do not capture the full antioxidant profile or the contribution of individual compounds. Therefore, the results should be interpreted as indicative of general antioxidant capacity and phenolic availability rather than a complete characterization of antioxidant activity.

#### Sensory properties of cookies

The results of the quantitative descriptive analysis (QDA) of cookies incorporating pomaces P1 (CP1), P2 (CP2) and P3 (CP3) are shown in Fig. 3.

**Fig. 3:**
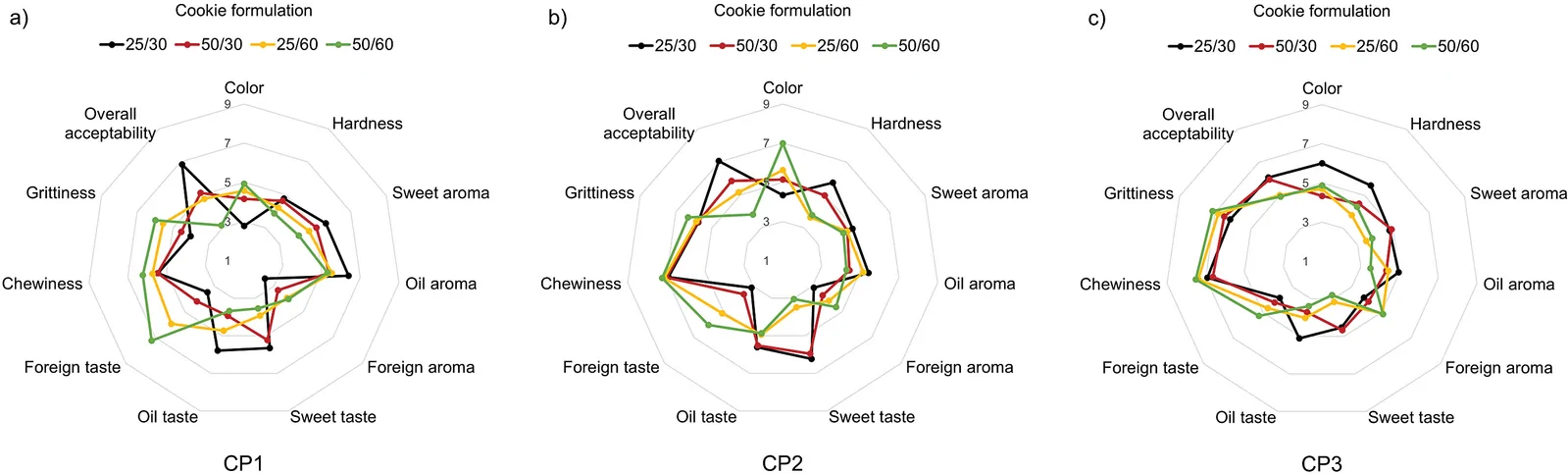
Quantitative descriptive analysis (QDA) of cookies formulated with: a) pomace 1 (CP1), b) pomace 2 (CP2), and c) pomace 3 (CP3). 25/30, 25/60, 50/30 and 50/60=cookies with reduced mass fractions of oil by 25 and 50 % and sugar by 30 and 60 %, respectively

Color was one of the attributes showing marked differences among formulations. For CP1 and CP2, the 50/60 formulation exhibited a significantly darker color than the others, with a progressive reduction in color observed as the pomace content decreased, as indicated by the ∆E* values previously reported (Table 4), which are perceptible to the human eye. In the case of CP3, 25/30 formulation was the darkest, following the trend observed and previously explained (Fig. 1).

Regarding hardness, cookies with lower sugar reductions (25/30 and 50/30) were the hardest, consistent with instrumental hardness results (Table 4). Sweetness was significantly higher in cookies with lower sugar reductions, regardless of the type of pomace added. Differences in oil taste were detected in some formulations with lower oil reductions, particularly between CP1 and CP2. Foreign taste showed significant differences in cookies, being greater in those formulations with the highest pomace content (50/60), reflecting the influence of both the type and quantity of pomace incorporated.

Other attributes, such as sweet aroma, oil aroma, foreign aroma, chewiness and graininess, did not show significant differences, although certain trends were observed. Formulations with higher pomace content (CP1 50/30, CP2 50/60 and CP3 50/60) tended to exhibit greater chewiness, graininess, and perception of foreign aroma or taste. Overall acceptability showed significant differences in CP1 and CP2, with formulations containing the highest sugar and oil reductions (25/60 and 50/60) receiving lower scores. For CP3, no significant differences were observed, although the 50/60 formulation was perceived as the least acceptable.

These results demonstrate that the type of pomace and the degree of sugar and oil reduction selectively influence sensory attributes and overall acceptability, allowing identification of formulations that best combine sensory attributes and functional value. In CP1 and CP2, formulations with moderate sugar and oil reductions (25/30 and 50/30) generally showed higher overall acceptability, preserving adequate sweetness and texture while limiting the perception of foreign taste associated with higher pomace amounts. In contrast, formulations with the highest pomace incorporation and greatest reduction (formulation 50/60) tended to have lower overall acceptability, mainly due to increased hardness, reduced sweetness, and greater foreign taste perception. For CP3, no significant differences in overall acceptability were observed, although the 50/60 formulation was consistently perceived as the least acceptable. This suggests that pomaces P1 and P2 may better tolerate higher amounts of incorporation. Taken together, formulations with intermediate pomace amounts and moderate sugar and oil reductions emerged as the most promising candidates for further optimization, combining improved nutritional quality with sensory attributes positively evaluated by trained panelists.

Notably, while sweetness was significantly lower in formulations with greater sugar reduction, non-nutritive sweeteners were not used to match amounts typically preferred by consumers. Instead, a gradual lowering of sweetness perception thresholds was applied, following Lingiardi *et al.* (*55*), who suggested that maintaining high sweetness intensity in reformulated products may reinforce consumer preference for highly sweet foods, thereby limiting the effectiveness of policies aimed at reducing daily sugar exposure. Thus, the reformulation strategy in this study was designed not only to improve the nutritional profile of the product but also to encourage sustainable, long-term changes in dietary patterns.

Finally, it should be acknowledged that the sensory evaluation was conducted with a limited number of trained panelists and did not include a consumer hedonic test. Therefore, conclusions regarding sensory evaluation should be interpreted as a description of the formulations according to the attributes evaluated, rather than as indicators of general consumer acceptance or preference, which should be confirmed in future studies involving a larger group of untrained consumers.

## CONCLUSIONS

Pomace incorporation, combined with reduced oil and sugar, proved to be an effective strategy for enhancing the nutritional quality of cookies with moderate technological and sensory changes within acceptable quality ranges. The use of whole fruit and vegetable pomaces increased dietary fiber while enabling substantial reductions in added sugar and fat. Moreover, the reformulated cookies exhibited high content of phenolic compounds and antioxidant capacity after *in vitro* digestion, indicating the potential bioaccessibility of bioactive components. These improvements were achieved while maintaining acceptable texture and appearance, as well as favorable sensory attributes, according to the evaluations by a trained sensory panel, supporting the technological feasibility of this reformulation approach. This descriptive sensory analysis should be complemented by future hedonic studies to assess consumer acceptability.

Beyond product quality, this strategy contributes to the sustainable use of food industry by-products, promoting circular economy practices and adding value to materials that are often underutilized. Overall, the combined reformulation approach represents a promising pathway for developing bakery products with enhanced nutritional attributes and reduced environmental impact, in line with current trends towards healthier and more sustainable food systems.

Future work should include a detailed characterization of the phenolic profiles of pomace to better understand the interactions and behavior of these compounds within the digested fractions, providing further insight into the mechanisms underlying their bioaccessibility.

## SUPPLEMENTARY MATERIAL

Supplementary materials are available at: https://www.ftb.com.hr/images/pdfarticles/2026/July-September/FTB-64-298-S1.pdf.

## Data Availability

Data are available from the corresponding author upon reasonable request.

## Appendix

**Table S1:** Attributes and reference values used in the sensory evaluation of cookies

Attribute	The lowest reference value	The highest reference value
Color	1=light (alfajor cookie layers FANTOCHE®)	9=dark (cookie “Chocolinas” ARCOR®)
Hardness (force required to fracture the material between the thumb and index finger)	1=low (cookie “Vainillas” MAURI®)	9=high (cookie without sugar, oil or pomace, with flavoring)
Sweet aroma	1=low (cookie without sugar, oil or pomace, with flavoring)	9=high (control cookie)
Oil aroma	1=low (cookie without sugar, oil or pomace, with flavoring)	9=high (control cookie)
Foreign aroma	1=low (cookie without sugar, oil or pomace, with flavoring)	9=high (pomace)
Sweet taste	1=low (cookie without sugar, oil, or pomace, with flavoring)	9=high (control cookie)
Oil taste	1=low (cookie without sugar, oil, or pomace, with flavoring)	9=high (control cookie)
Foreign taste	1=low (cookie without sugar, oil, or pomace, with flavoring)	9=high (pomace)
Chewiness (number of bites required for the product to be ready to swallow)	1=low (alfajor cookie layers FANTOCHE®)	9=high (cookie “Muesli” MURKE®)
Graininess (perception of particles when pressed between the tongue and the palate)	1=low (alfajor cookie layers FANTOCHE®)	9=high (cookie “Muesli” MURKE®)
Overall acceptability (harmony level of all mentioned parameters)	1=low	9=high
